# An integrated analysis of three medulloblastoma clinical trials refines risk-stratification approaches for reducing toxicity and improving survival

**DOI:** 10.1093/neuonc/noaf250

**Published:** 2025-10-24

**Authors:** Kyle S Smith, Sandeep K Dhanda, Catherine A Billups, Edgar Sioson, Congyu Lu, Airen Zaldivar Peraza, Karishma Gangwani, Yimei Li, Qian Li, Tong Lin, Jeff M Michalski, Roger J Packer, James M Olson, Sarah E S Leary, Maryam Fouladi, Amar Gajjar, Xin Zhou, Arzu Onar-Thomas, Paul A Northcott, Giles W Robinson

**Affiliations:** Department of Developmental Neurobiology, St. Jude Children’s Research Hospital, Memphis (K.S.S., P.A.N.); Center of Excellence for Neuro-Oncology Sciences (CENOS), St. Jude Children’s Research Hospital, Memphis (K.S.S., P.A.N.); Division of Neuro-Oncology, Department of Oncology, St. Jude Children’s Research Hospital, Memphis (S.K.D., A.G., G.W.R.); Department of Biostatistics, St. Jude Children’s Research Hospital, Memphis (C.A.B., Y.L., Q.L., T.L., A.O.-T.); Department of Computational Biology, St. Jude Children’s Research Hospital, Memphis (E.S., C.L., A.Z.P., K.G., X.Z.); Department of Computational Biology, St. Jude Children’s Research Hospital, Memphis (E.S., C.L., A.Z.P., K.G., X.Z.); Department of Computational Biology, St. Jude Children’s Research Hospital, Memphis (E.S., C.L., A.Z.P., K.G., X.Z.); Department of Computational Biology, St. Jude Children’s Research Hospital, Memphis (E.S., C.L., A.Z.P., K.G., X.Z.); Department of Biostatistics, St. Jude Children’s Research Hospital, Memphis (C.A.B., Y.L., Q.L., T.L., A.O.-T.); Department of Biostatistics, St. Jude Children’s Research Hospital, Memphis (C.A.B., Y.L., Q.L., T.L., A.O.-T.); Department of Biostatistics, St. Jude Children’s Research Hospital, Memphis (C.A.B., Y.L., Q.L., T.L., A.O.-T.); Department of Radiation Oncology, Washington University School of Medicine, St Louis (J.M.M.); Brain Tumor Institute, Gilbert Family Neurofibromatosis Institute, Center for Neuroscience and Behavioral Medicine, Children’s National Hospital, Washington (R.J.P.); Ben Towne Center for Childhood Cancer and Blood Disorder’s Research, Seattle Children’s Research Institute, Seattle (J.M.O., S.E.S.L.); Department of Pediatrics, University of Washington, Seattle (J.M.O., S.E.S.L.); Fred Hutchinson Cancer Research Center, Seattle (J.M.O., S.E.S.L.); Ben Towne Center for Childhood Cancer and Blood Disorder’s Research, Seattle Children’s Research Institute, Seattle (J.M.O., S.E.S.L.); Department of Pediatrics, University of Washington, Seattle (J.M.O., S.E.S.L.); Fred Hutchinson Cancer Research Center, Seattle (J.M.O., S.E.S.L.); Nationwide Children’s Hospital, Columbus (M.F.); Division of Neuro-Oncology, Department of Oncology, St. Jude Children’s Research Hospital, Memphis (S.K.D., A.G., G.W.R.); Department of Computational Biology, St. Jude Children’s Research Hospital, Memphis (E.S., C.L., A.Z.P., K.G., X.Z.); Department of Biostatistics, St. Jude Children’s Research Hospital, Memphis (C.A.B., Y.L., Q.L., T.L., A.O.-T.); Department of Developmental Neurobiology, St. Jude Children’s Research Hospital, Memphis (K.S.S., P.A.N.); Center of Excellence for Neuro-Oncology Sciences (CENOS), St. Jude Children’s Research Hospital, Memphis (K.S.S., P.A.N.); Division of Neuro-Oncology, Department of Oncology, St. Jude Children’s Research Hospital, Memphis (S.K.D., A.G., G.W.R.)

**Keywords:** craniospinal irradiation, medulloblastoma, methylation, molecular ­categorization, risk stratified therapy

## Abstract

**Background:**

The identification of clinical and molecular heterogeneity in medulloblastoma has produced risk-stratified therapy, but establishing the most effective yet least toxic regimens has remained elusive owing to numerous treatment options. To improve risk-stratification, we performed an integrated analysis from three clinical trials.

**Methods:**

Medulloblastoma patients from ACNS0331/NCT00085735, ACNS0332/NCT00392327, and SJMB03/NCT00085202 were included if they had methylation profiling. Molecular groups [WNT, SHH, Group 3 (G3), and Group 4 (G4)], subgroups, and copy number variations were procured from methylation profiles and mutations from next-generation sequencing. Data was assembled into an interactive portal to capture patient characteristics. Cross-trial comparisons, univariable, and multivariable analyses were conducted and used to derive a risk-stratification schema.

**Results:**

Eight hundred ninety-eight patients (WNT = 131, SHH = 151, G3 = 220, G4 = 396) were included. Progression-free-survival (PFS) distributions among analogous cross-trial cohorts were not different, demonstrating no survival advantage of any one therapy over another. The addition of carboplatin to high-dose craniospinal irradiation (HDCSI) containing regimen was selectively superior in PFS in G3/G4 subgroup 3 (*P* = 0.048) and G3/G4 subgroup 2 (*P* = 0.035) to HDCSI regimens without carboplatin. Nine actionable risk-stratified groups were identified consisting of 2 WNT groups (low, high-risk), 3 SHH groups (low-, average-, very-high-risk), and 4 G3/G4 groups (low-, average-, high-, and very-high-risk).

**Conclusions:**

Our integrated cross-trial analysis suggests toxicity can be reduced by eliminating disproportionate differences in therapy in favor of a more uniform treatment backbone. Moreover, we propose and model a risk-classification system that identifies the most appropriate cohorts on which to trial significant dose reductions in craniospinal irradiation or select treatment intensifications.

Key Points

1. The outcomes of children with medulloblastoma are strongly influenced by the heterogeneity of the disease and the treatment received.
2. Improvements in survival and morbidity will result once risk factors are properly aligned to the least toxic and most effective regimens.

Importance of the Study

The biggest conundrum surrounding medulloblastoma therapy is that survival is, predominantly, predicated on higher craniospinal irradiation doses and augmented chemotherapy while reductions in therapy engenders a better quality of life. In this study, we integrated clinical and molecular data from three of the largest North American medulloblastoma clinical trials. This allowed us to compare outcomes by treatment differences across many variables on a much larger scale than ever before. Consequently, we were able to devise and model a novel risk-stratification algorithm that proposes therapeutic de-escalation to 40% of the population, reduced toxicity to 45% through a more moderate backbone of standard therapy, and augmented therapy to only 15% without compromising survival. In addition to providing a structure for next-generational clinical trial practice, this study deposits its data into an easily accessible interactive web-based portal for all to explore.

Medulloblastoma (MB) is among the most common pediatric central nervous system (CNS) malignancies affecting 6 in 1,000,000 children worldwide.^1^ Multimodal therapy, which combines surgery, craniospinal irradiation (CSI), and chemotherapy, has brought the cure rate to approximately 75%.^2-6^ However, survivors experience detrimental consequences from the therapy received. Cognitive deficits, hearing loss, infertility, neuropathy, frailty, endocrinopathies, and heightened risk for premature death are just some of the late effects that afflict this population.^7–10^ Still, not all regimens used to treat MB are the same, and substantial differences are present in the dose and modality of CSI, the size of the radiation boost area, the doses of chemotherapy used, the number of cycles given, and the definition of what constitutes high-risk or average-risk disease.

Adding to this complexity is the molecular heterogeneity. MB divides into 4 molecular groups: wingless/INT1-activated (WNT); sonic hedgehog-activated (SHH); and two non-WNT/non-SHH (NWNS) groups called group 3 (G3) and group 4 (G4).^4^^,^^11^^,^^12^ Additionally, characteristic molecular features, such as chromosomal copy-number variations (CNVs), gene amplifications, and mutations have been implicated as being prognostic even within the groups.^2^^,^^13-16^ For example, the presence of *MYC* amplification in NWNS MB, *MYCN* amplification, *GLI2* amplification, or *TP53* mutation in SHH MB all portend a very poor prognosis.^2^^,^^13^^,^^16-21^ In contrast, WNT MB and NWNS MB with specific whole chromosome aberrations exhibit excellent prognoses.^16^^,^^22^^,^^23^ More recently, the molecular groups have undergone additional subcategorization into four subgroups of SHH (SHH-1, SHH-2, SHH-3, SHH-4) and eight subgroups of NWNS tumors (G3/G4 subgroup 1-8), among which, some are also prognostic.^1^^,^^2^^,^^4^^,^^15^^,^^24-26^ Risk-stratification schemes, which integrate whole chromosome aberrations and subgroups, have been developed and validated for NWNS MB.^25^^,^^26^

While treatment modifications are appealing, providers hesitate to change because dose-reductions can fail, leading to increased relapse rates, meaning more intensive treatment, or worse, loss of lives that would otherwise have been saved. This hard lesson was learned in three recent trials and has justifiably placed any proposed dose reduction under scrutiny. The ACNS0331 study (NCT00085735), which randomized children 3-7 years-old with average-risk MB to low-dose CSI (LDCSI) versus standard-dose CSI (SDCSI) with identical chemotherapy regimens, reported inferior outcomes in the LDCSI group.^5^ A pilot study that omitted radiation therapy in children with average-risk WNT-MB found an unexpectedly high relapse rate.^27^ Likewise, a study that treated children with average-risk WNT MB with focal conformal radiation, omitting the cranio-spinal portion, and adjuvant chemotherapy terminated prematurely due to relapses.^28^

Nevertheless, progress has come in the form of combining molecular characteristics and clinical characteristics to risk stratify therapy in a more measured way. Three recent trials SJMB12 (NCT01878617), ACNS1422 (NCT02724579), and PNET5 (NCT02066220) explored a moderate reduction of CSI from 23.4 Gy SDCSI to 15-18 Gy LDCSI for average-risk WNT MB patients. While results are not yet published, conference abstract presentations and the continuation of these closely monitored studies over many years without early termination suggest that this strategy has not been appreciably inferior. Hence, these studies have set a precedent that an effective way to risk-stratify MB patients is by stratifying therapy using molecular and clinical risk.

However, because the WNT population accounts for only 10-15% of the MB population, this progress feels modest. Even now, throughout North America and Australia, the treatment regimens from three older MB trials form the basis of therapy for most patients. These three prospective trials include ACNS0331 for average-risk MB, ACNS0332 (NCT00392327) for high-risk MB, and SJMB03 (NCT00085202) for both average and high-risk MB. ACNS0331 demonstrated the non-inferiority of reduced field radiation boost to tumor bed compared to whole posterior fossa, but demonstrated inferiority of LDCSI in young children with average-risk MB.^5^ ACNS0332 demonstrated the superiority of chemoradiotherapy augmented with carboplatin for high-risk Group 3 MB, but no benefit of carboplatin in other high-risk MB groups, and no benefit of augmented maintenance therapy with isotretinoin.^6^ SJMB03 found ERBB2 status did not predict survival, but suggested improved risk-stratification through the integration of clinical and molecular risk factors.^2^

The outcomes of these MB trials were published with extensive array of clinical and molecular data.^2^^,^^5^^,^^6^ However, due to disease heterogeneity and subtle differences in trial eligibility, a thorough comparison of results has remained challenging. We saw this as an opportunity, and pooled and assembled the data into analogous categories that facilitate contrast and comparison. We sought to understand what components within the regimens are indispensable for survival and what are omittable or reducible such that toxicities can be minimized. In addition, we sought to use the resultantly large and meticulously annotated cohort to test the relevance of putative risk factors and model the results on outcome.

Additionally, we built the information into an interactive data portal [ https://viz.stjude.cloud/st-jude-childrens-research-hospital/visualization/medulloblastoma-integrative-analysis-portal∼2882] that allows the research community to query complex multi-dimensional data to investigate outcomes by therapy received. This pooled data and accompanying portal represent a resource to assist in generating hypotheses for subsequent trial design and, in our estimation, will ultimately result in improved therapy and outcomes for children with MB.

## Methods

### Study Cohort

The study cohort comprised patients enrolled in ACNS0331, ACNS0332, and SJMB03. Institutional review board approval was obtained at each institution that participated in the trials and written informed consent was obtained for all participants.

ACNS0331, run by the Children’s Oncology Group (COG), was a multi-institutional phase III clinical trial for patients 3-21 years old newly diagnosed with average-risk MB that enrolled from April 2004 to January 2014. Patients were post-operatively reviewed for eligibility. Average-risk was defined by not having large-cell/anaplastic histology (LC/A), no evidence of metastatic disease (M0), and no evidence of residual tumor of > 1.5 cm^2^ (R0). Patients aged 3-7 years were randomized to two treatment modifications: (1) to SDCSI of 23.4 Gy or LDCSI of 18 Gy; and (2) to receive whole posterior fossa radiation therapy (PFRT) or involved field radiation therapy (IFRT) to total dose of 54 Gy. Patients aged 8-21 years received SDCSI and were randomized to PFRT vs IFRT. The clinical target volume (CTV) margin for PFRT was the whole posterior fossa and for IFRT was 1.5 cm around the post-operative bed. Planned treatment included vincristine 1.5 mg/m^2^ IV once weekly on weeks 2-7 during radiotherapy. After chemoradiotherapy and a 4-week rest, patients received 9 cycles of alternating maintenance chemotherapies (cycle A and cycle B) delivered in an AABAABAAB pattern. Cycle A was 6 weeks and consisted of lomustine 75 mg/m^2^ PO once on day 1; vincristine 1.5 mg/m^2^ IV once daily (max dose 2.0 mg) on days 1, 8, and 15; and cisplatin 75 mg/m^2^ IV once on day 1. Cycle B was 4 weeks and consisted of cyclophosphamide 1000 mg/m^2^ IV once daily on days 1 and 2; and vincristine 1.5 mg/m^2^ IV once daily (max dose 2.0 mg) on days 1 and 8.

SJMB03 was a multi-institutional phase III non-randomized clinical trial for patients 3-21 years old with newly diagnosed MB, supratentorial primitive neuroectodermal tumor (PNET), or atypical teratoid rhabdoid tumor that enrolled from September 2003 to June 2013. Patients with MB were post-operatively stratified into two risk groups. Average-risk was defined by M0 and R0 status. High-risk was defined by either evidence of metastatic disease (M+) as indicated by MRI of the brain or spine and/or cytological examination of the CSF, and/or residual tumor of > 1.5 cm^2^ (R+). Patients with average-risk disease were treated with SDCSI (23.4 Gy) followed by IFRT (total dose 55.8 Gy). Patients with high-risk disease received high dose CSI (HDCSI - 36-39.6 Gy) followed by IFRT (total dose 55.8-59.4 Gy) to the primary tumor and additional RT (total dose 50.4-59.4 Gy) to areas of macroscopic metastatic disease (> 0.5 cm). The CTV margin was 1.0 cm for both risk groups. After radiation and a 6-week rest, patients received four 4-week cycles of vincristine 1.0 mg/m2 IV once daily (max dose 2.0 mg) on day −4 and +6, cisplatin 75 mg/m^2^ IV once daily on day −4, and cyclophosphamide 2000 mg/m^2^ IV once daily on day −3 and −2 with autologous stem cell rescue on day 0.

ACNS0332, run by COG, was a multi-institutional phase III clinical trial for patients 3-21 years old newly diagnosed with high-risk MB and PNET that enrolled from March 2007 to September 2018. Patients were post-operatively reviewed for eligibility. High-risk was defined by LC/A histology, M+, or R+. Patients underwent 2 treatment randomizations: (1) HDCSI with weekly vincristine with or without daily carboplatin and (2) 6 cycles of maintenance chemotherapy with or without 12 cycles of isotretinoin during and following maintenance. Patients received 36-39.6 Gy HDCSI followed by PFRT (total dose 55.8 Gy) and additional RT (total dose 50.4-55.8 Gy) to areas of macroscopic metastatic disease (> 0.5 cm). All patients received vincristine 1.5 mg/m^2^ IV once weekly on weeks 1-6 during radiotherapy. Those randomized to carboplatin, received 35 mg/m^2^ IV once daily for a total of 30 doses during radiotherapy. After chemoradiotherapy and a 6-week rest, patients received six 4-week cycles of maintenance chemotherapy with or without isotretinoin. A maintenance cycle consisted of cisplatin 75 mg/m^2^ IV once daily on day 1, vincristine 1.5 mg/m^2^ IV once daily (max dose 2.0 mg) on days 1 and 8, and cyclophosphamide 1000 mg/m^2^ IV once daily on days 1 and 2. Those randomized to receive isotretinoin, received 80 mg/m2 PO once daily on days 1, 16-28 during each maintenance cycle and 80 mg/m^2^ PO once daily on days 15-28 for cycles 7-12.

The primary endpoints assessed in these studies were PFS, Event-Free Survival (EFS), and Overall Survival (OS).

### Data Portal

The purpose of the data portal was to provide a comprehensive, user-friendly point-and-click interface for exploring integrated datasets. Demographic, clinical, and genomic data were organized as variables into a data dictionary and placed on an interactive web-based portal. Within, variables can be selected, combined, analyzed and visualized by multiple plots consisting of: (1) **summary plots** that visualize the distribution of a given variable; (2) **survival plots** to visualize outcome by select variables; (3) **sample matrix plots** that provide graphical representation of the overall annotations and genomic alterations of the samples; (4) **scatter plots** that visualize the tumor clustering patterns based on methylomic state; (5) **genome browser** plots that display a lollipop map on the gene of all the mutations found across a user-adjustable cohort.

### Molecular Data

Molecular data includes mutation data, cytogenetics profiles, and molecular groups as reported in the trial publications.^2^^,^^5^^,^^6^ Significant focal events (amplifications and deletions) were identified from 450K/EPIC methylation arrays using conumee v1.2.0 and were defined as focal (<10 Mb) with deviations of more than four times the Median Absolute Deviation (MAD) from their reference baseline. Molecular subgroups were determined by previously described methods.^15^

### Statistical Analyses

Progression-free-survival was defined as the time interval from date on study to date of relapsed, or progressive disease, or death from any cause, or to the date of last follow-up. Outcome distributions were estimated using the method of Kaplan-Meier (KM). The log-rank test was used to compare outcome distributions among groups. Cox regression models were used to examine risk factors in SHH and G3/G4 subjects. The association between categorical variables was assessed using Fisher’s Exact test, while the impact of multiple independent variables on survival outcomes was analyzed via the Cox Proportional Hazard model. Model comparison and performance were evaluated using bootstrap-based approaches, by PFS, by concordance statistics, and by area under the curve (AUC)/ receiver operating characteristic (ROC) curves (**Supplementary Results**). Molecular graphics and analyses were performed with Chimera.

## Results

### Cross-Trial Comparison Discriminates Essential from Excessive Therapy

**Figure 1.**
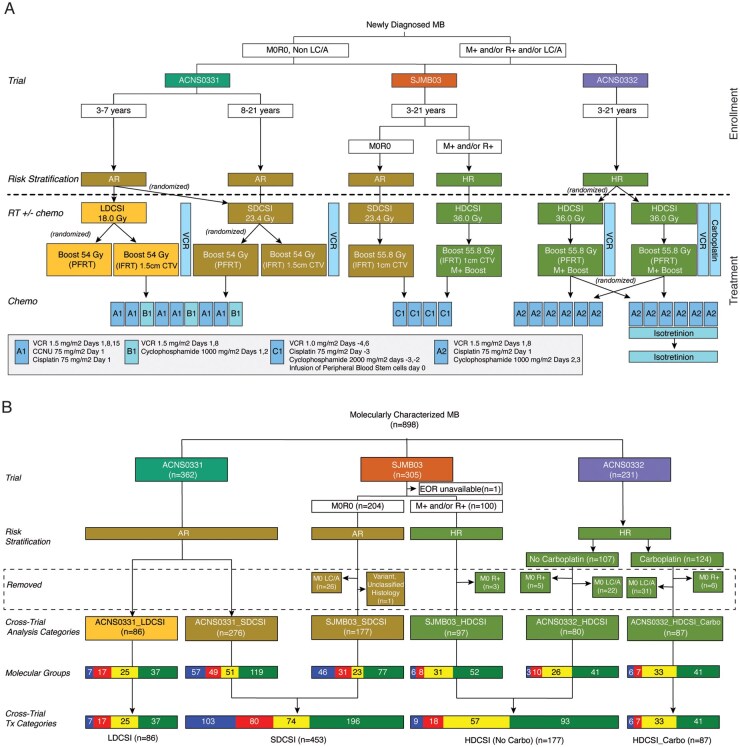
Overview of the 3 trials and categorization of patients into groups for comparative analysis. (A) Compares the distribution of newly diagnosed medulloblastoma (MB) patients, the risk stratification, and the therapy by trial. ACNS0331 enrolled patients 3-21 years old with non-metastatic (M0) non-residual (R0) and non LC/A pathology; SJMB03 enrolled patients 3-21 years old with M0, metastatic (M+) disease, R0, and residual (R+) disease regardless of pathology; ACNS0332 enrolled patients 3-21 years old with LC/A pathology, R+ disease, or M+ disease. While these regimens appear similar, there are differences. M0 LC/A patients all received HDCSI on ACNS0332 whereas they received SDCSI on SJMB03. ACNS0331 and ACNS0332 prescribed chemo-radiation therapy (vertical side bars), with weekly vincristine during radiation therapy and with randomization to daily carboplatin to high-risk patients, whereas SJMB03 therapy did not. SJMB03 treated all patients (average and high-risk) with an IFRT Boost of radiation with a CTV of 1 cm, whereas ACNS0331 randomized to IFRT Boost CTV of 1.5 cm versus whole posterior fossa Boost (PFRT), and ACNS0332 used a PFRT. The Boost RT dose on SJMB03 and ACNS0332 was 55.8 Gy and greater than the 54 Gy given on ACNS0331. SJMB03 treated patients with high-dose cyclophosphamide-containing regimens that used autologous stem cell harvest and rescue (C1). ACNS0331 and ACNS0332 prescribed 6 doses of cisplatin (A1 and A2), whereas SJMB03 prescribed 4 (C1). (B) Distributes molecularly characterized patients into 6 “Cross-trial Analysis Categories” and removes patients whose data is inconsistent with the groupings (ie M0 LC/A and M0 R+ patients). The second to last row labeled “Molecular Groups” shows the distribution of molecular groups within each categories. The final row merges categories into 4 “Cross-trial Treatment Categories” and shows the distribution of the molecular groups in order to explore outcomes across the 4 major therapeutic differences. AR, Average Risk; CTV, clinical target volume; EOR, extent of resection; HDCSI, high-dose craniospinal irradiation; IFRT, involved field radiation therapy; LC/A, large cell/anaplastic histology; LDCSI, low-dose craniospinal irradiation; HR, high risk; M0, nonmetastatic; M+, metastatic; R0, no residual tumor; R+ residual tumor; PFRT, posterior fossa radiation therapy; VCR, vincristine.

The distribution of MB patients, risk stratification, and the therapy administered on the three trials is summarized in **Figure 1A**. Of 1,055 patients enrolled in the trials, 898 had tumor samples which were methylation profiled, formulating the study cohort. Among these, 754 had next-generation DNA sequencing (609 WES—tumor only, 129 paired tumor/germline WES, 16 paired WGS). A matrix display of the entire cohort by consensus molecular group summarizes the characteristic clinical and molecular features (**Supplementary Figure S1A**). Dimensionality reduction and visualization of DNA methylation array data illustrates the distribution of molecular groups (**Supplementary Figure S1B**) and subgroups (**Supplementary Figure S1C and D**), respectively.

To facilitate a cross-trial comparison, 803 (89%) of the 898 were placed into six categories (**Figure 1B**): (1) ACNS0331_LDCSI—patients with M0R0, non-LC/A MB treated with LDCSI on ACNS0331 (*n* = 86); (2) ACNS0331_SDCSI—patients with M0R0 non-LC/A MB treated with SDCSI on ACNS0331 (*n* = 276); (3) SJMB03_SDCSI—patients with M0R0 non-LC/A MB treated with SDCSI on SJMB03 (*n* = 177); (4) SJMB03_HDCSI—patients with M+ MB treated with HDCSI on SJMB03 (*n* = 97); (5) ACNS0332_HDCSI—patients with M+ MB treated with HDCSI on ACNS0332 (*n* = 80); (6) ACNS0332_HDCSI_Carbo—patients with M+ MB treated with HDCSI and concurrent carboplatin with radiation on ACNS0332 (*n* = 87). The remaining 95 patients were comprised of SJMB03 MB patients with M0 LC/A histology treated with SDCSI, ACNS0332 MB patients with M0 LC/A histology treated with HDCSI, and SJMB03 M0R+ and ACNS0332 M0R+ MB patients treated with HDCSI. These patients were excluded from the cross-trial analysis; however, their data were used in subsequent analyses and remain available within the portal.

Proportionally, ACNS0332 enrolled fewer WNT patients and more G3 patients than ACNS0331 and SJMB03 (**Figure 1B****; Supplementary Figure S2**). However, when divided into the 6 analogous groups there were no significant differences in molecular group distributions between patients who received SDCSI and patients who received HDCSI on the different trials (**Supplementary Figure S2B**). Similarly, when restricted to ages between 3-7 years, there was no difference between the proportions of the molecular groups who received LDCSI or SDCSI (**Supplementary Figure S2C**).

**Figure 2.**
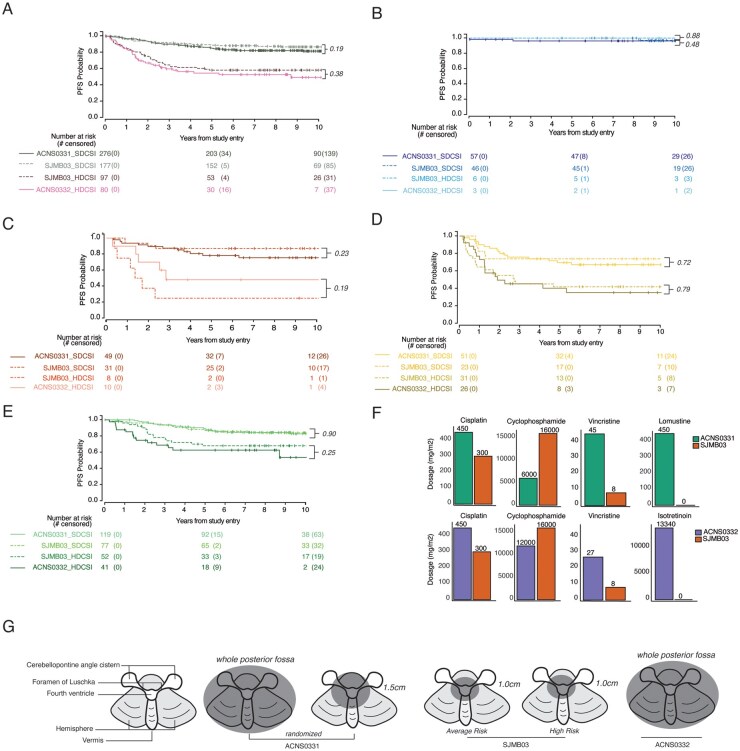
Comparison of the 4 Cross-trial Analysis Categories containing SDCSI regimens (ACNS0331_SDCSI and SJMB03_SDCSI) and HDCSI regimens without carboplatin (ACNS0332_HDSCI and SJMB03_HDCSI). No significant PFS differences between the SDCSI patients from ACNS0331 and SJMB03 or the HDCSI patients from SJMB03 and ACNS0332 were observed when viewed as: (A) the entire cohort without incorporating any molecular features, (B) WNT, (C) SHH, (D) Group 3, and (E) Group 4. (F) Compares the cumulative doses of chemotherapy drugs utilized across all three trials. (G) Compares the clinical target volume (CTV) utilized across all three trials.

To evaluate the outcomes of similar populations treated on different trials, the PFS of ACNS0331_SDCSI and SJMB03_SDCSI were compared, as was the PFS of SJMB03_HDCSI and ACNS0332_HDCSI. Comparison was performed first without incorporating any molecular features and then by each of the four molecular groups. No significant survival differences were observed in the SDCSI regimens or in the HDCSI regimens (**Figure 2A-E**).

In **Figure 2F**, the cumulative dose per body surface area (mg/m^2^) of the chemotherapy agents used in the three trials are summarized. Despite equivalent outcomes, ACNS0331 prescribed 6 times the amount of vincristine and ACNS0332 prescribed 3 times more vincristine than SJMB03 therapy. SJMB03 prescribed 2.5 times the amount of cyclophosphamide and ACNS0332 prescribed 2 times more cyclophosphamide than ACNS0331 therapy. ACNS0331 and ACNS0332 prescribed 1.5 times more cisplatin than SJMB03. Furthermore, the CTV of radiation varied from 1 cm to whole posterior fossa boost across the three trials with no impact on survival (**Figure 2G****)**.

### Cross-Trial Treatment Category Analysis Identifies the Impact of Treatments according to Molecular Identity

Due to similar PFS outcomes, the SDCSI cases (ACNS0331_SDCSI + SJMB03_SDCSI) and the HDCSI cases (ACNS0332_HDCSI + SJMB03_HDCSI) were combined into 2 large cohorts [SDCSI, *n* = 453; HDCSI (No Carbo) *n* = 177 **Figure 1B**] for a more robust analysis to estimate the difference in outcome between LDCSI vs SDCSI, and HDCSI with carboplatin vs. HDCSI without carboplatin.

To investigate the outcome of LDCSI relative to SDCSI as an aggregate and by molecular group, the PFS of ACNS0331_LDCSI (hereon LDCSI, *n* = 86) was compared to all patients who received SDCSI. Inferior PFS was observed in the LDCSI group when compared to the SDCSI group in the entire cohort without incorporating molecular features (*P* = 0.003), in G4 (*P* = 0.05), in G3 (*P* = 0.04), and no difference in PFS was observed between the LDCSI and SDCSI in the WNT or SHH molecular groupings (**Supplementary Figure S3A-E)**. However, when the comparison was restricted by age 3-7 years, the difference in PFS by molecular group was no longer significant (**Supplementary Figure S4B-E**), even in G4 which differed from the ACNS0331 study finding.^5^ These findings emphasize that stratification by molecular group to LDCSI is inadequate as a strategy for risk determination, and continue to caution that, when broadly applied, LDCSI therapy remains inferior to SDCSI.

**Figure 3.**
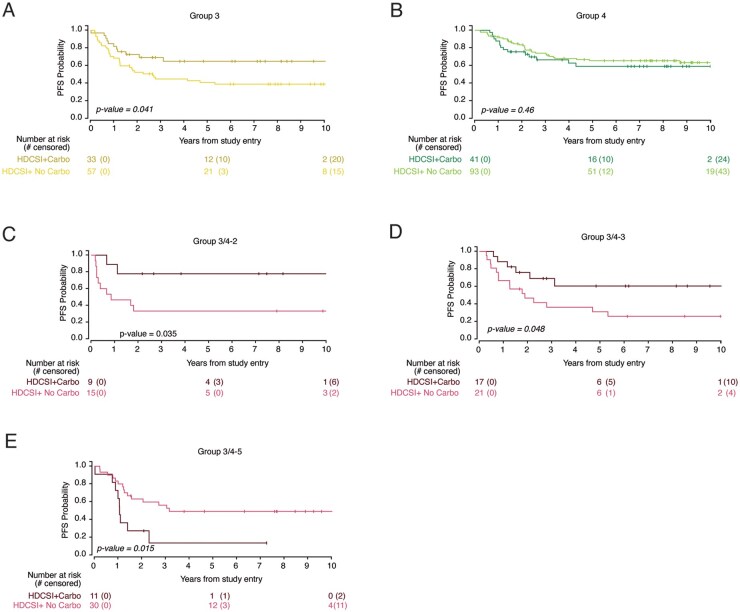
Comparison of cross-trial treatment categories containing HDCSI (light line) to HDCSI_Carbo (dark line). Superior survival was observed for HDCSI_Carbo patients relative to HDCSI patients in (A) G3 patients, *P* < 0.05, (C) G3/G4-2 patients, *P* < 0.05, and (D) G3/G4-3 patients, *P* < 0.05. No significant PFS differences were observed in (B) G4. Inferior survival was observed in HDCSI_Carbo patients relative to HDCSI patients in (E) G3/G4-5 patients.

To evaluate the effect of adding carboplatin during radiation to M+ patients, the PFS of ACNS0332_HDCSI_Carbo (hereon HDCSI_Carbo, *n* = 87) was compared to all M+ MB patients who received HDCSI without carboplatin (ACNS0332_HDCSI + SJMB03_HDCSI). No difference in PFS was observed in the aggregate cohort, or in WNT, and SHH (**Supplementary Figure S5A-C**). Consistent with the ACNS0332 study findings,^6^ an improvement in PFS was observed in G3 patients who received carboplatin during radiation over those M+ patients who did not (*P* = 0.041, **Figure 3A**) but not in G4 patients (*P* = 0.46, **Figure 3B**). However, given a larger dataset, G3/G4 molecular subgroups were evaluated identifying an improvement in PFS in G3/G4-2 (*P* = 0.035; **Figure 3C**) and 3 (*P* = 0.048; **Figure 3D**) treated with carboplatin, but not in subgroups 1, 4, 6, 7, or 8 (**Supplementary Figure S6A-E**). Curiously, the PFS in G3/G4-5 treated with carboplatin was worse than without (*P* = 0.015; **Figure 3E**). The relevance of improved PFS in G3/G4-2 and G3/G4-3 should not be underestimated since these have been reported to be the most aggressive subgroups within G3, often enriched for MYC amplifications.^2^^,^^15^^,^^24^ This finding, suggesting a selective benefit to patients diagnosed with particularly aggressive tumors, represents a notable refinement to what was previously reported on ACNS0332 that warrants evaluation in future clinical trials.

### Harmonization across Trial Datasets Implicates Risk-Factors and Stimulates a Novel Risk-Stratification Approach

Recent studies have suggested multiple risk classification algorithms using a variety of combined clinical-and-molecular approaches.^2^^,^^20^^,^^22^^,^^25^^,^^26^^,^^29^ We sought to examine these in this harmonized dataset to see if we could delineate a more precise model focused on clinically actionable risk-stratification. Here, we excluded the WNT cases for their known favorable prognosis and separated the cohorts into SHH and G3/G4. To limit the effect of treatment-related bias, we restricted the initial analysis to patients who did not receive LDCSI (SHH and G3/G4) or HDCSI_Carbo (G3/G4) and performed both univariable and multivariable analyses of putative risk factors across the remaining cohorts.

**Figure 4.**
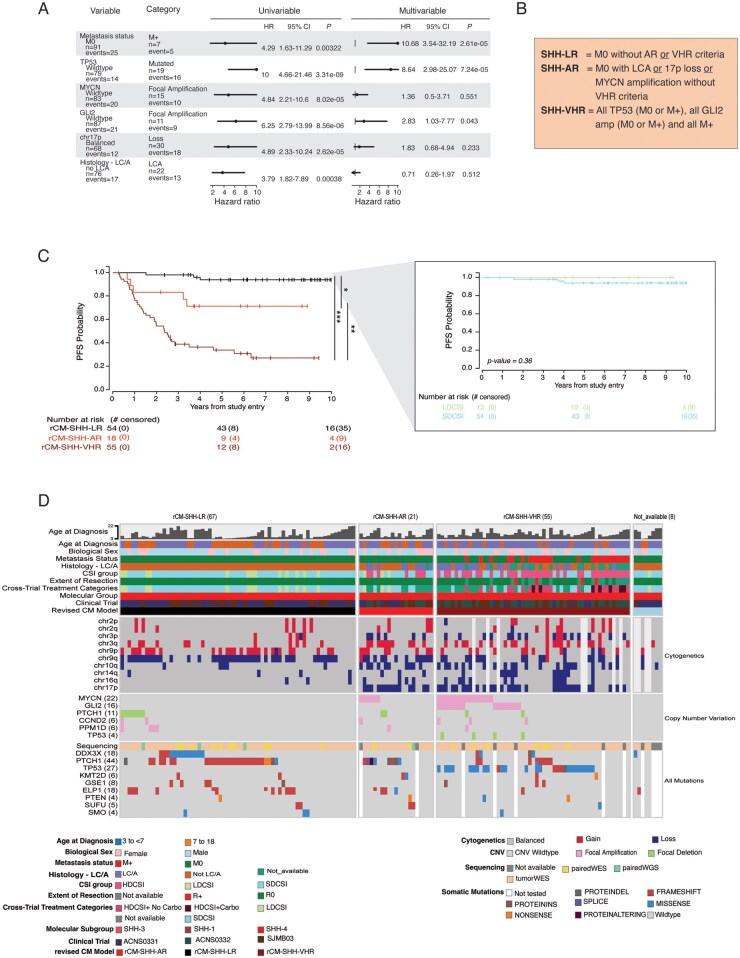
Exploring molecular and clinical risk in the SHH cohort. (A) Univariable and Multivariable analysis of prognostic features (B) Clinical-molecular risk category assignment (C) Kaplan-Meier PFS plot by revised clinical-molecular risk categories showing significant differences in PFS among the 3 groups. Pop-out plot shows no PFS difference between the rCM-SHH-LR patients who received LDCSI and SDCSI (D) Oncoprint showing clinical and molecular information from 151 patients in the SHH cohort. Cases are organized by the revised clinical molecular risk categories in the following order: rCM-SHH-LR, rCM-SHH-AR, rCM-SHH-VHR, Not available.

Univariable analyses of the SHH cohort showed *TP53* mutation, *MYCN* amplification, *GLI2* amplification, 17p loss, LC/A histology, and M+ were each associated with poor prognosis. Multivariable analysis demonstrated *TP53* mutation, *GLI2* amplification, and M+ status were statistically significant predictors of poor prognosis (**Figure 4A**). Considering these risk factors with the intent to stratify clinical outcome resulted in 3 risk groups: a SHH low-risk group (SHH-LR; no high-risk features), a SHH average-risk group (SHH-AR; LC/A, *MYCN* amplification, and/or 17p loss), and a SHH very-high-risk group (SHH-VHR; *TP53* mutation or *GLI2* amplification or metastatic disease) (**Figure 4B-D****)**. Subsequently, we identified the SHH-LR patients from the LDCSI cohort and compared the PFS of this group to the SHH-LR group who received SDCSI. No difference in PFS was observed, suggesting that LDCSI is a viable strategy to pursue for these patients (**Figure 4C****)**. No improvement in survival for SHH-VHR was seen across trial, by CSI dose, by different CTVs, or with carboplatin but small sample sizes limit more definitive conclusions.

**Figure 5.**
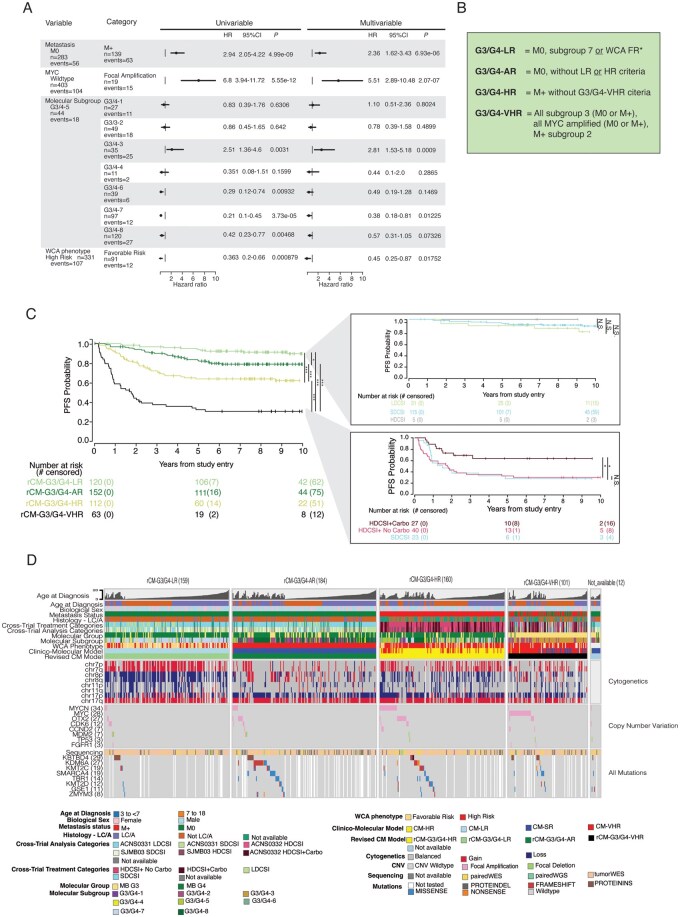
Exploring molecular and clinical risk in the G3/G4 cohort. (A) Univariate and Multivariate analysis of prognostic features (B) Clinical-molecular risk category assignment (C) Kaplan-Meier PFS plot by revised clinical-molecular risk categories showing significant differences in PFS among the 4 groups. Upper pop-out plot shows no PFS difference between the rCM-G3/G4-LR patients who received LDCSI and SDCSI. Lower pop-out plot shows a significantly superior PFS for r-CMG3/G4-VHR patients who received HDSCI_Carbo over those who did not. (D) Oncoprint showing clinical and molecular information from 616 patients in the G3/G4 cohorts. Cases are organized by the revised clinical molecular risk categories in the following order: rCM-G3/G4-LR, rCM-G3/G4-AR, rCM-G3/G4-HR rCM-G3/G4-VHR, Not available.

Univariable analyses of the combined G3 and G4 cohort showed *MYC* amplification, M+, Group 3, and G3/G4-3 each associated with a poor prognosis while G3/G4-6, -7, -8, and whole chromosome aberration favorable risk (WCA FR) phenotype (consisting of at least 2 of chromosome 7 gain, chromosome 8 loss, and 11 loss)^22^ each associated with a favorable prognosis. Notably, *MYCN* amplification was not associated with poor prognosis, validating prior studies.^20^^,^^22^ LC/A was not evaluated as a risk factor due to many absent histology calls limiting sample size. Multivariable analysis demonstrated that G3/G4-3, M+ status, and *MYC* amplification were significantly associated with poor prognosis and G3/G4-7 and WCA FR phenotype were associated with favorable PFS (**Figure 5A**). Modeling of these risk factors to stratify clinical outcome and accommodating for the previously observed poor outcome of G3/G4-2 when metastatic, resulted in 4 risk groups: a G3/G4-LR group (M0 and G3/G4-7 or WCA FR phenotype), a G3/G4 average-risk (AR) group (M0 without LR or VHR criteria), a G3/G4-HR group (M+ without VHR criteria) and a G3/G4-VHR group [all G3/G4-3 (M0 or M+), all MYC amplified (M0 or M+), M+ G3/G4-2] **(****Figure 5B and C****)**. Subsequently, we compared the PFS between G3/G4-LR patients who received SDCSI to those who received LDCSI. No difference in PFS was observed, which although limited in power, suggests that LDCSI represents a viable option for these patients. Also, we compared the PFS outcomes between G3/G4-VHR patients who received HDCSI_Carbo to those who received HDCSI-No_Carbo and observed a significant improvement in 5-year PFS from <35% to >65% (*P* = 0.005) (**Figure 5C**). Pertinently, no difference in PFS was observed between G3/G4-HR patients who did and did not receive carboplatin (**Supplementary Figure S7**).

Lastly, we compared our proposed model, named the revised-clinico-molecular (rCM) model, to previously considered risk-stratification models using bootstrap-based approaches to evaluate reproducibility, by PFS to compare outcome prediction, and by examining Harrell’s concordance statistics and AUCs/ROC curves to estimate model performance. Comparator models included the clinical-molecular approach from Mynarek et al,^25^ the continuum approach from Williamson et al,^29^ the PNET5 approach,^30^ and a previous approach proposed in Gajjar et al.^2^ By comparison, the performance of our rCM model was similar to other models (**Supplementary Figures S8 and S9 and Supplementary Results**).

## Discussion

In this study we observed that the harmonization of the clinical and molecular data from 3 of the largest, simultaneously conducted and recently-published MB clinical trials identified: (1) the components of therapy that appeared to be the most necessary and those which could be substantially reduced or even omitted; (2) the risk factors that were the most prognostic and thus the highest priority for future clinical action; and (3) groups that could benefit from major treatment modifications (ie radiation reduction or addition of carboplatin as a radiation sensitizer) and groups that would not.

Important caveats are that, while the comparisons of the trials are very helpful in postulating that patients receive excessive treatment, this exercise does not reveal the optimal regimen or dose; instead, it simply provides contrast. This is especially important to consider before adjusting various components within regimens, given that the higher dosing of one component in one regimen may be compensating for lower dosing of another component in the same regimen. Therefore, implementation of treatment changes based on these results are warranted only if used to support a hypothesis in a well-designed, carefully monitored clinical trial, because, if implemented without proper oversight, recipients will be at risk of serious harm.

Nevertheless, when the relevant trial cohorts were standardized to include only clinically and molecularly matched patients who received SDCSI with adjuvant chemotherapy and patients who received just HDCSI with adjuvant chemotherapy (ie, without carboplatin chemoradiation), there was no difference in survival. The absence of any survival difference was maintained across the molecular groups and was observed despite the treatment differences between the trials. Patients who were prescribed 6-fold the dose of vincristine had similar survival to patients who did not; patients who received a 1 cm CTV boost had similar survival to those who received a 1.5 cm CTV boost or a whole posterior fossa boost; survival of patients who received high doses of cyclophosphamide (16 g/m^2^) and autologous stem cell rescue was not different to survival of patients who received between 6 and 12 g/m^2^ of cyclophosphamide and no autologous stem cell rescue; and patients who were cumulatively prescribed greater than 300 mg/m^2^ cisplatin did not have improved survival over patients who were prescribed 300 mg/m^2^. Consequently, this represents an opportunity to broadly reduce therapy to a “new standard” regimen, whereby, 1 cm CTV or less is used for boost dosing, vincristine therapy is limited to reduce neuropathy, cisplatin usage is capped to reduce hearing loss, and cyclophosphamide dose is limited to reduce the risk of infertility. The SJMB12 trial (NCT01878617) has taken this approach and limited the CTV to 0.5 cm, the cumulative dose of vincristine to 8 mg/m^2^, cisplatin to 300 mg/m^2^, and cyclophosphamide to 12 g/m^2^. The results of this study will determine if this approach produces similar outcomes while reducing side effects. Furthermore, it could advance a more tolerable backbone of therapy that would better allow the incorporation of novel therapies.

While risk factors have been explored across many MB studies, the accuracy and precision of the results have been limited by small cohort size and incomplete data. In contrast, this large dataset, with its comprehensive clinical and molecular collections, offers an unprecedented opportunity to explore the many putative risk factors while controlling for differences in treatment and molecular features.

For WNT patients, as expected, we observed favorable responses across all treatment regimens, including those with LDCSI, SDCSI, HDCSI, and HDCSI with Carboplatin. In contrast to a recent report,^31^ we found no significant difference in PFS between patients receiving the higher cumulative dose cyclophosphamide regimen (SJMB03) vs. the lower (ACNS0331), however, none were prescribed less than 6 g/m^2^. Moreover, we did not find any new prognostic features that would support intensification to any subset of this population. This substantiates the current approaches evaluating LDCSI in the non-metastatic population (SJMB12, NCT01878617; PNET5, NCT02066220; ACNS1422, NCT02724579; FOR-WNT2, NCT04474964) and encourages the moderate de-intensification approach being evaluated in the metastatic population in PNET5.^30^

In agreement with other published works, we found SHH disease to be heterogenous, and its prognosis to be strongly influenced by several risk factors.^2^^,^^13^^,^^20^ Robust analysis showed that these risk factors are not independent and, often, co-occur. Multivariable analysis showed that very poor prognosis is principally driven by three main features: *TP53* mutations, *GLI2* amplifications, and M+ disease. In fact, when any are present then the outcome is abysmal and only a quarter survive. Uniquely, we found that *MYCN* amplification, which has been widely cited to harbor a poor prognosis,^4^^,^^13^^,^^17^^,^^18^^,^^20^^,^^32^^,^^33^ was not associated with poor prognosis on a multivariable analysis, but rather, only when seen in conjunction with any of the other 3 features. While intriguing, this finding should be cautiously interpreted and requires validation, given that *MYCN* amplifications were called from methylation array data and not FISH, most often overlap with high-risk features and rarely occur in isolation. Moreover, we observed a highly favorable SHH group, lacking unfavorable features, that has excellent survival even when treated with LDCSI. Taken together, this information allows for the risk-stratification of SHH patients into 3 clinically-actionable groups that should be examined on a future clinical trial: the SHH-LR group for which LDCSI should be explored; the SHH-AR group for which SDCSI should still be given to patients with isolated *MYCN* amplifications or LC/A histology; and the SHH-VHR group for which novel regimens are desperately needed.

Similarly, we found G3/G4 disease to be heterogeneous, and prognosis to be strongly influenced by several risk factors. Favorable prognosis was associated with molecular subgroup G3/G4-7 or WCA FR phenotype and very poor prognosis was associated with molecular subgroup G3/G4-3, *MYC* amplifications, or molecular subgroup G3/G4-2 with M+ disease. This allowed us to develop a risk-stratification method akin to recently published clinico-molecular approaches,^25^^,^^26^ with some nuanced modifications. This method, separates G3/G4 into 4 clinically-actionable groups: the G3/G4-LR group for which LDCSI should be explored; the G3/G4-AR group for which SDCSI should still be given to non-metastatic patients even with isolated *MYCN* amplifications or LC/A histology; the G3/G4-HR group for which HDCSI and adjuvant chemotherapy should still form the backbone of therapy; and G3/G4-VHR group for which a significant survival advantage is predicted if these patients are treated with HDCSI_Carbo regimens.

**Figure 6.**
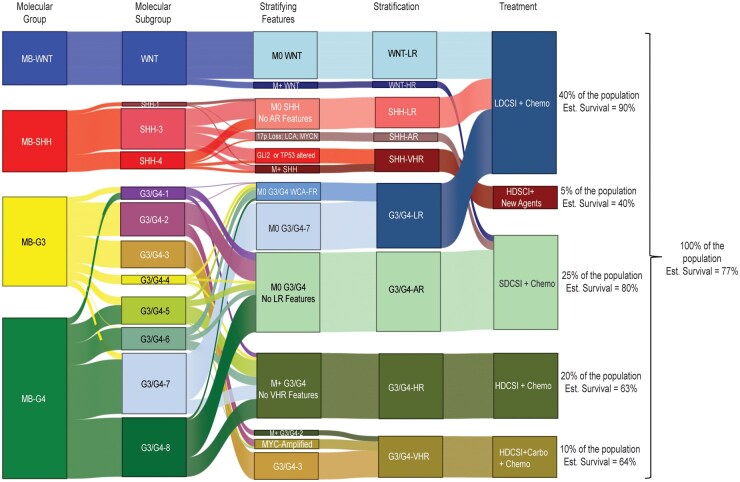
A Sankey diagram illustrating how to deploy the new risk-stratification algorithm. By starting with molecular group, progressing through molecular subgrouping, incorporating major stratifying features (ie metastatic status, MYC amplifications) medulloblastoma patients can be stratified into the 9 revised clinical-molecular groupings (WNT-LR, SHH-LR…) and then sorted into 5 treatment categories. In this way a much greater proportion of the population than ever before would be treated with LDCSI, HDCSI with carboplatin would reach the subset of the population that stands to benefit most, and the remaining patients would benefit from a more rigorous stratification system justifying the choice of therapy and receive chemotherapy that is modified to eliminate unnecessary dosing.

**Figure 6** depicts a treatment algorithm based on this rCM approach and shows the clinical impact of such a design. By this approach, (1) 40% of the population will be treated with a LDCSI regimen and are expected to retain or exceed 90% survival; (2) 25% will be treated with SDCSI and are expected to retain >80% survival; (3) 20% will receive HDCSI and are expected to retain >60% survival; and (4) 10% will receive HDCSI_Carbo with a resultant >60% survival. Together, this leaves only 5% of patients in dire need of novel therapy with new agents to improve on an estimated 40% survival. Altogether, survival is estimated be maintained at the current level of 75%, or better, and given that this approach would deploy adjuvant chemotherapy that conforms to our “new standard”, the quality of life would be significantly improved for all patients.

Although this study comprises one of the largest collections of molecularly defined MB enrolled in clinical trials, it has limitations related, mostly, to clinical trial design and the inherent heterogeneity of MB. Regarding clinical trial design, none of the trials mandated a central review of imaging or histology to determine each trial’s risk-stratification. Up to 8% inaccurate staging by imaging has been reported in medulloblastoma trials,^5^^,^^34^^,^^35^ and a lack of uniform reporting of histology prevented us from broadly assessing the effect of histologic variants across this integrated cohort. The trials did not comprehensively collect toxicity data. This is unfortunate as it prevents direct toxicity comparison across regimens. However, since the toxicities of therapy (CSI, vincristine, cisplatin, cyclophosphamide) are widely documented and increase with cumulative dose,^7^^,^^36–38^ it is reasonable to suggest that lowering the cumulative dose will decrease toxicity. LDCSI was only given to 3-7-year-old patients, thus preventing evaluation of LDCSI in older patients. While there is no known difference in survival between an older or a younger child within the same molecular subgroup, this distinction could not be evaluated in this study and age merits an evaluation in future studies. Also, carboplatin was only given as a radiosensitizer in the ACNS0332 trial, and validation in more MB patients, particularly within G3, is needed.

Concerning heterogeneity, most participants lacked germline DNA sequencing, preventing us from effectively investigating the association of pathogenic cancer predisposition with outcome. Future studies must address this knowledge gap in sufficiently sized, clinically annotated cohorts, especially for hallmark predisposition genes such as *TP53*, and novel risk genes like *ELP1*.^21^^,^^39^^,^^40^ Finally, and importantly, the analysis resulted in small sample sizes for many variables and our results must be interpreted with caution. Specifically, the lack of a statistically significant difference often occurs because of inadequate sample size and should not be interpreted as definitive evidence of absence of effect.

Ways to improve sample size include adding more cohorts such as those from European and Asian studies. Additionally, inclusion of currently maturing studies will be highly informative, to compare the effects of these newer approaches to those presented here. These include PNET5, that is prescribing LDCSI to WNT and using carboplatin with CSI^30^; SJMB12, which is exploring LDCSI in WNT, a lower-dose cyclophosphamide than SJMB03, and pemetrexed and gemcitabine in NWNS patients; and ACNS1422, which is exploring LDCSI in WNT.

In anticipation and to facilitate exploration of these data alongside other cohorts, we have deposited all of the data within this manuscript into an interactive publicly available data portal for all to investigate.

In conclusion, this study shows the practice-changing potential that comes from aggregating and analyzing meticulously curated datasets from clinical trials. Through this exercise we unearthed opportunities to minimize unnecessary toxicities, reduce neurocognitive-damaging therapy, and hone specific therapy to refractory disease; all the while preserving and bettering survival. Likewise, we believe that more findings will emerge if molecular and clinical data from other clinical trials are similarly evaluated, and we hope that this study will serve as a valuable resource that assists the entire community in accessing the necessary information needed to advance more effective therapy for children with medulloblastoma.

## Supplementary Material

noaf250_Supplementary_Data

## Data Availability

All data within this manuscript are available on an interactive publicly available data portal [https://viz.stjude.cloud/st-jude-childrens-research-hospital/visualization/medulloblastoma-integrative-analysis-portal∼2882]. Additionally, raw data from genome, exome, and methylation testing are being submitted to dbGAP and will be available at the time of publication.
