# Supplementary material for: An integrated analysis of three medulloblastoma clinical trials refines risk-stratification approaches for reducing toxicity and improving survival
Source: Neuro Oncol. 2025 Oct 24;28(1):268–81. doi: 10.1093/neuonc/noaf250 (PMC12962632; doi:10.1093/neuonc/noaf250)
Supplement: noaf250_Supplementary_Data [file noaf250_supplementary_data.pdf]

Figure S1

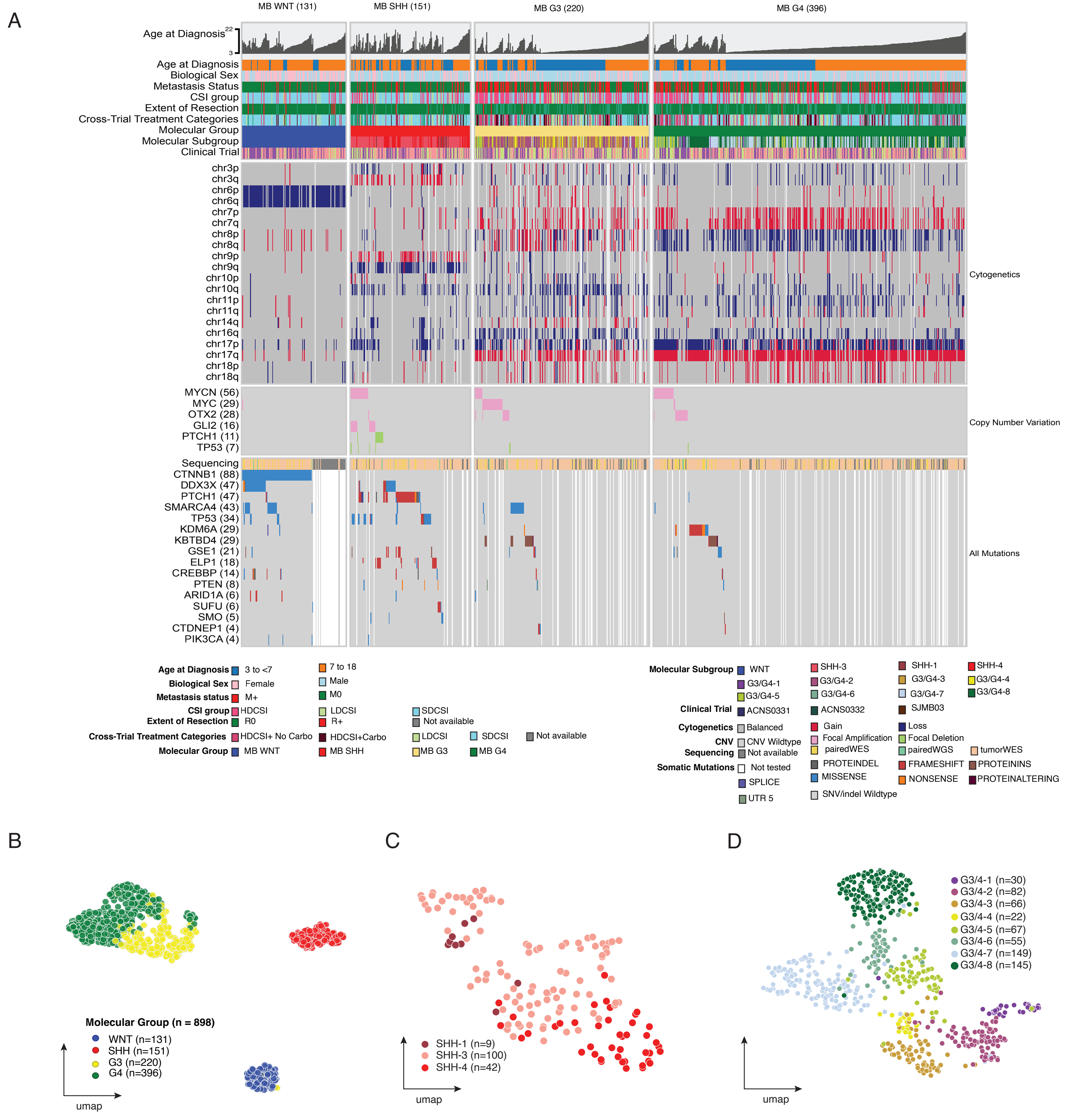

Supplementary Figure S1: Molecular breakdown of the entire cohort. (A) Oncoprint showing clinical and molecular information from of 898 patients who had methylation profiled tumor samples. Cases are organized from left to right by molecular group in the following order WNT, SHH, Group 3, Group 4. Alterations identified by tumor sequenc-ing are also shown for 754 samples (B) Umap plot of the 4 molecular groups illustrating molecular similarities and differences by clustering. (C) Umap plot of the 4 SHH molecular subgroups. (D) Umap plot of the 8 G3/G4 molecular subgroups.

Figure S2

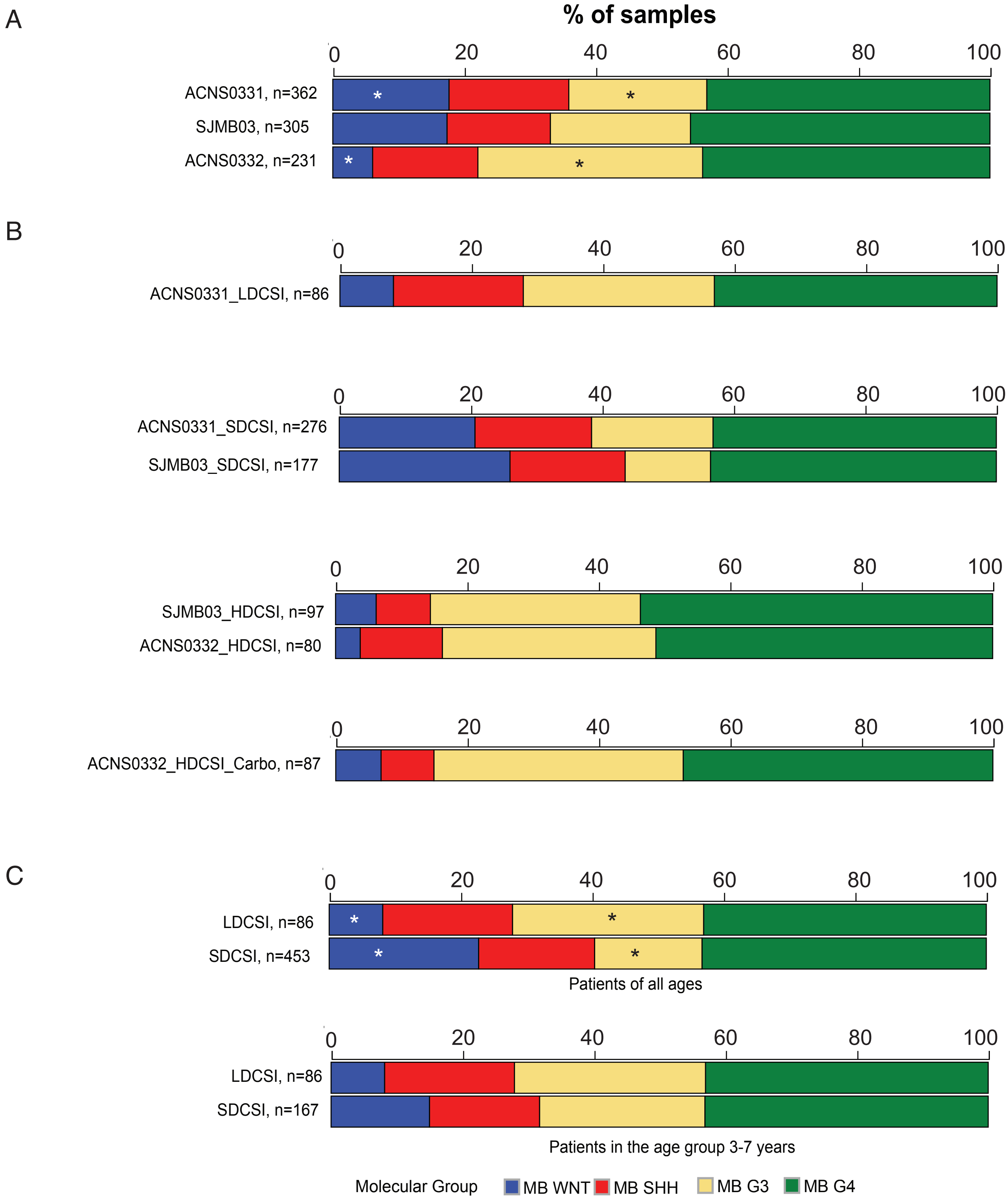

Supplementary Figure S2: Distribution of molecular groups across various studies and comparable groups based on cranio-spinal dosages and/or age categories. (A) The ACNS0332 study had a lower proportion of WNT patients and a higher proportion of G3 patients compared to ACNS0331 and SJMB03. (B) There were no significant differences in the proportions of molecular groups when comparing LDCSI (top panel), SDCSI (second panel), HDCSI (third panel), and HDCSI_carbo (fourth panel) across different studies. (C) Upper panel: Although fewer WNT and more G3 patients received LDCSI compared to those who received SDCSI, (Lower panel) the difference was not significant when considering patients aged 3-7.

Figure S3

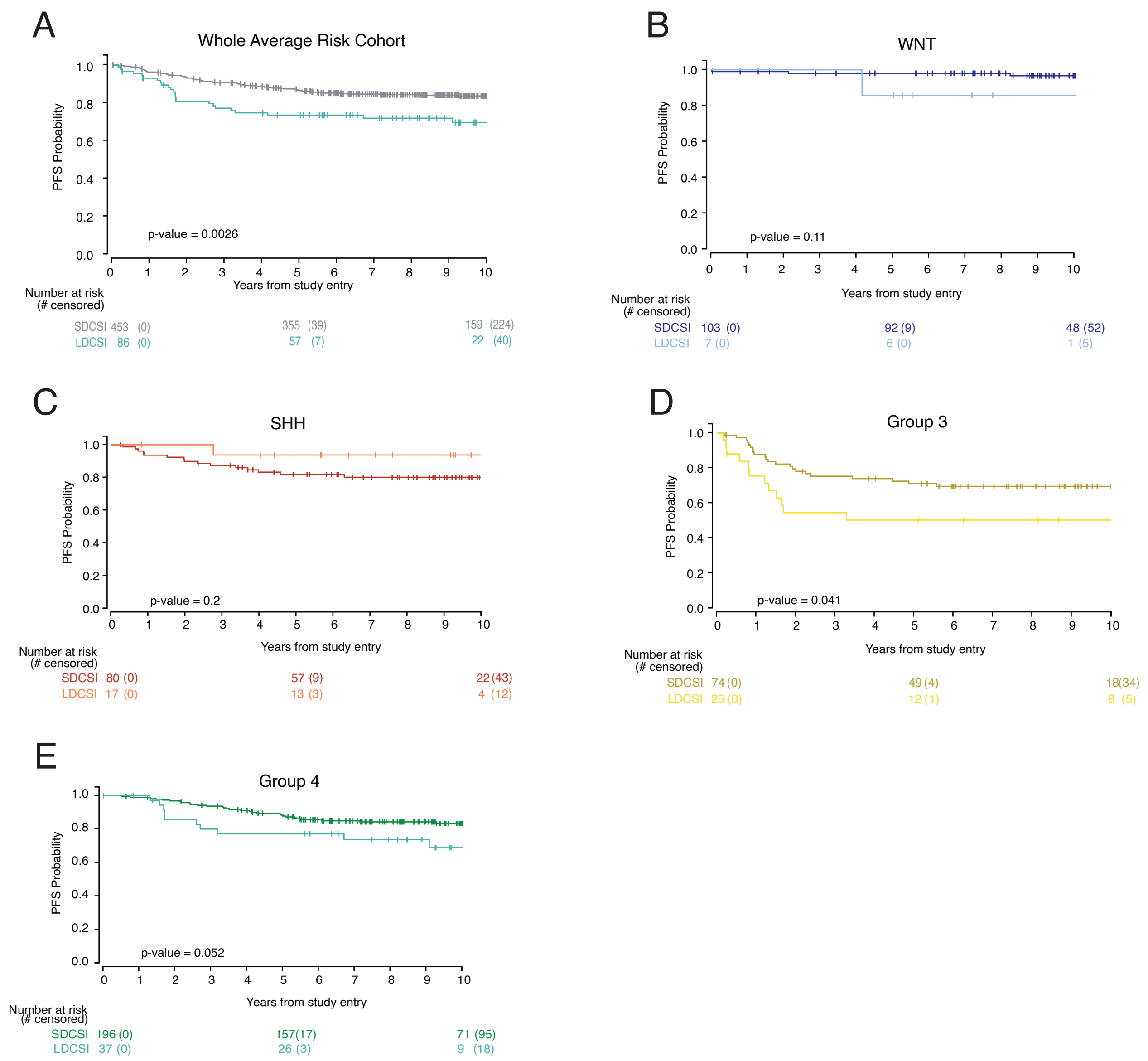

Supplementary Figure S3: Evaluation of LDCSI PFS relative to SDCSI PFS for all age patients. Inferior survival was observed when PFS of LDCSI for average risk (M0R0 non-LCA) patients was compared to average risk patients who received SDCSI across (A) the whole average risk cohort, $p<0.01$ (D) group 3, $p<0.05$ (E) group 4, $p=0.05$. No difference in PFS was observed for (B) WNT or (C) SHH patients receiving either LDCSI or SDCSI.

Figure S4

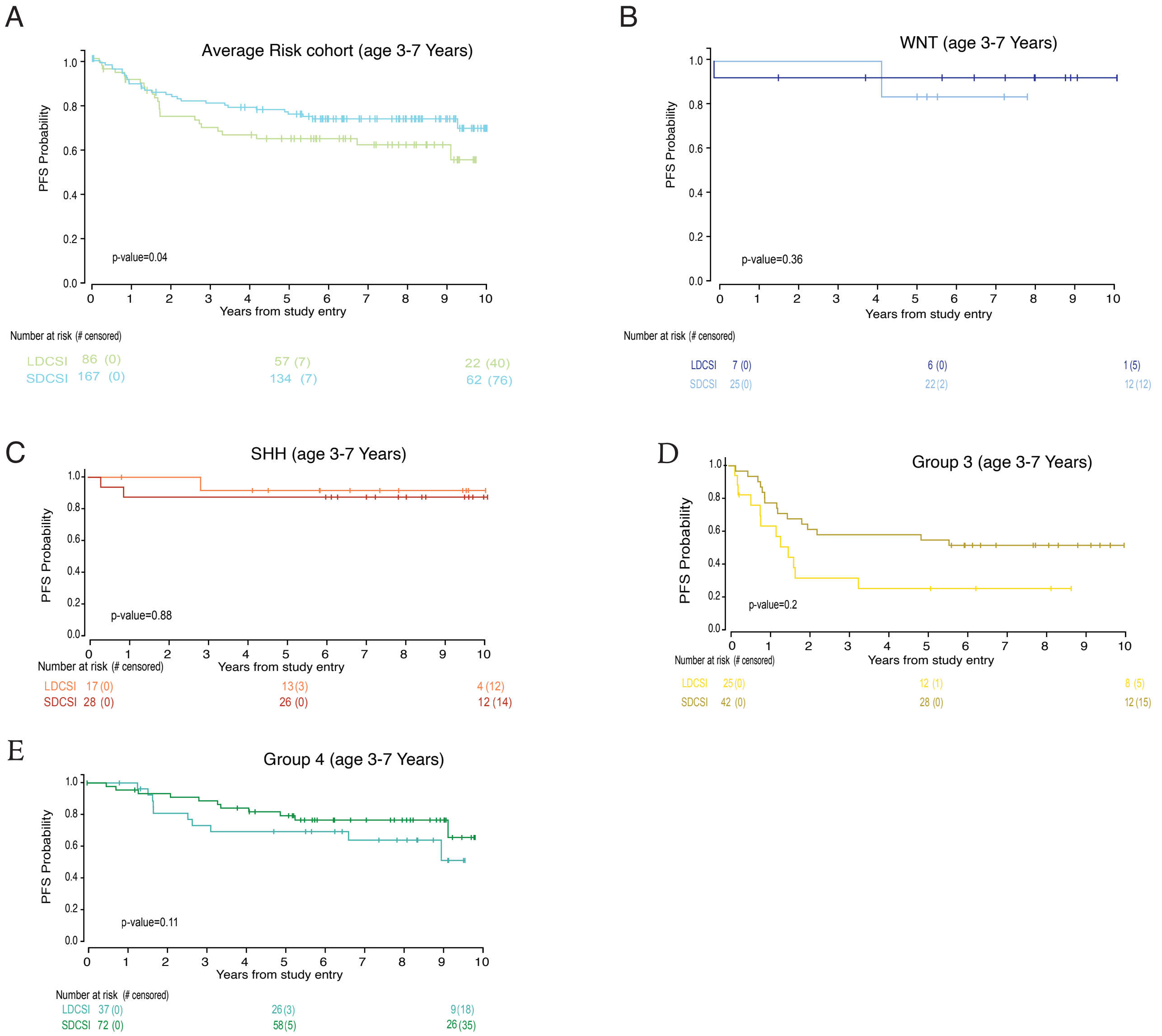

Supplementary Figure S4: Evaluation of LDCSI PFS relative to SDCSI PFS for age group 3-7 years. Inferior survival was observed when PFS of LDCSI for average risk (M0R0 non-LCA) patients was compared to average risk patients who received SDCSI across (A) the whole average risk cohort. No difference in PFS was observed for (B) WNT or (C) SHH or (D) Group 3 or (E) Group 4 patients receiving either LDCSI or SDCSI.

Figure S5

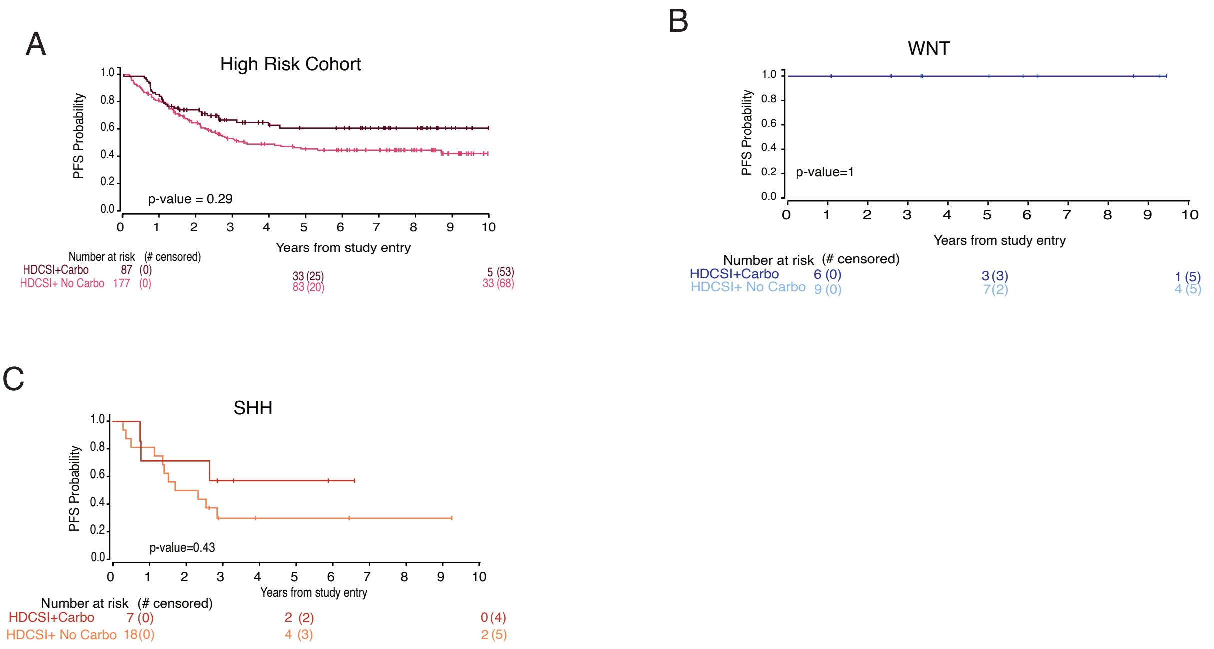

Supplementary Figure S5: Comparison of cross-trial treatment categories containing HDCSI to HDCSI_Carbo. No difference in PFS was observed in (A) the aggregate cohort, or in (B) WNT, and (C) SHH.

Figure S6

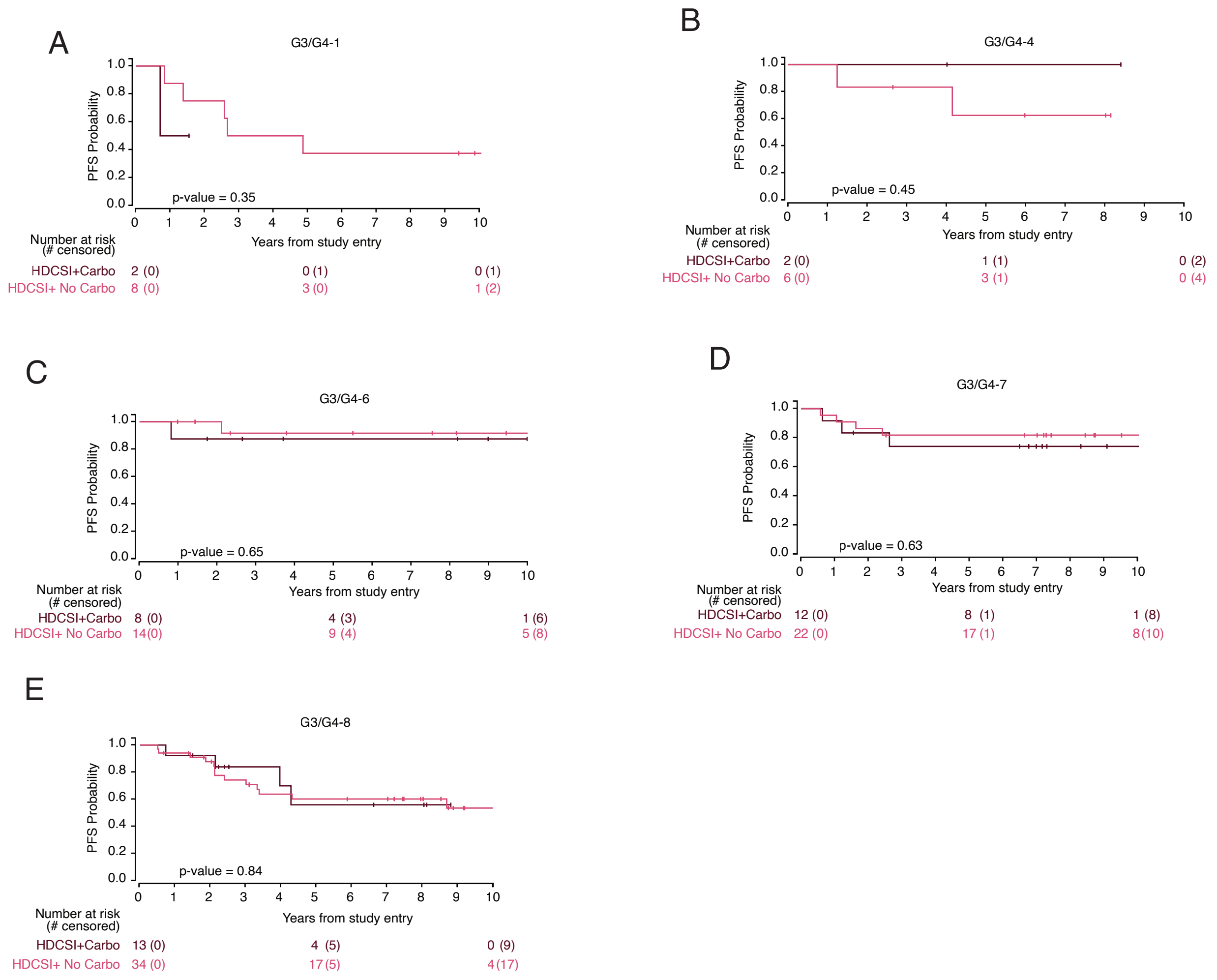

Supplementary Figure S6: Comparison of cross-trial treatment categories containing HDCSI (light line) to HDCSI_Carbo (dark line). No significant PFS differences were observed in (A) G3/G4-1, (B) G3/G4-4, (C) G3/G4-6, (D) G3/G4-7 and (E) G3/G4-8.

Figure S7

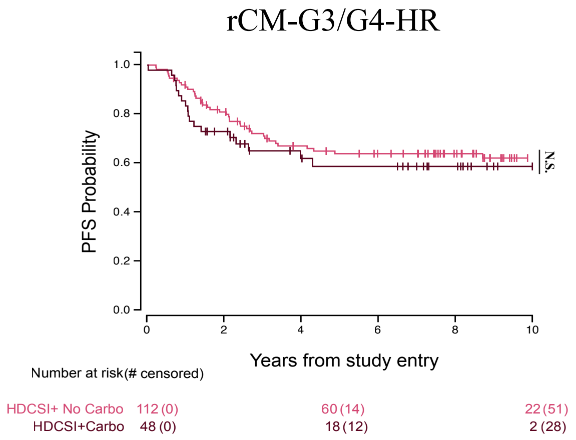

Supplementary Figure S7: Kaplan Meier graph showing no difference in PFS between the rCM-G3/G4-HR patients that received HDSCI with carboplatin (HDCSI+Carbo; darker red) and those that received HDCSI without carboplatin (HDCSI+ No Carbo; lighter red).

Figure S8

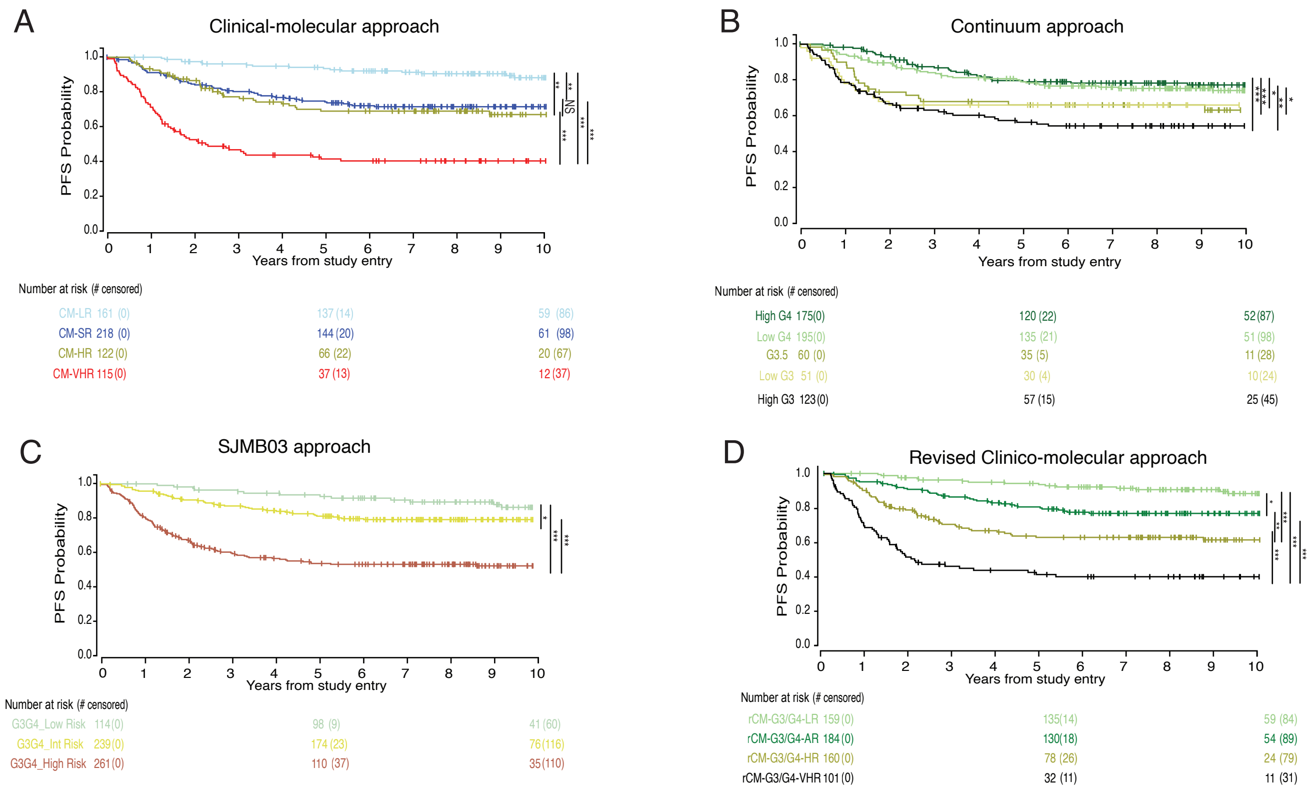

Supplementary Figure S8: Comparison of Progression Free Survival by four different G3/G4 risk-stratification methods. (A) Clinico-molecular approach, (B) Continuum approach, (C) SJMB03 approach, and (D) Revised Clinico-molecular approach proposed in this study.

Figure S9

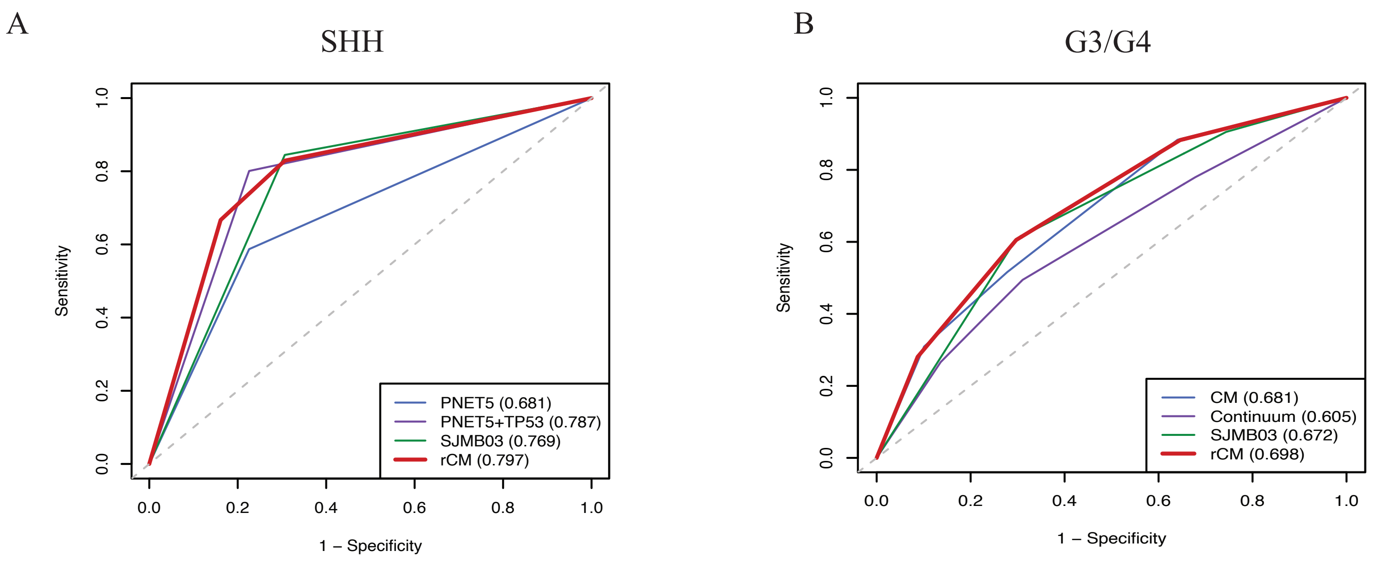

Supplementary Figure S9: ROC plots showing performance of different risk-stratification models relative to random performance (diagonal dotted line). A) Compares the model performance in the SHH cohort where rCM = solid red line, PNET5 = solid blue line, PNET5 + TP53 = solid purple line, SJMB03 = solid green line. B) Compares the model performance in the G3/G4 cohort where rCM = solid red line, CM = solid blue line, Continuum = solid purple line, SJMB03 = solid green line. In both plots the rCM model attained the highest AUC value. Abbreviations: rCM, revised clinico-molecular approach; CM, clinicomolecular approach; ROC, receiver operating characteristic.

Supplementary Results

Bootstrap-based approaches:

To evaluate the repeatability of our model vs. other risk stratification approaches published previously within the SHH and G3/G4 cohorts separately, from each molecular cohort we randomly selected (with-replacement) 1000 samples of the same size as the original cohort. In each bootstrap sample, we examined PFS by the various risk stratification models (SHH: rCM, SJMB03 approach, PNET5 approach and PNET5+TP53 approach;
G3/G4: CM, rCM, SJMB03 and Continuum approach) and summarized below the frequency of p-values <0.05 for the overall comparison across risk groups (global log-rank test) and for the ordered pairwise comparisons between risk groups. Separately, to evaluate the stability of the model, we randomly selected 1000 subsamples of 80% the data without replacement, stratified on event indicator to select 80% of patients with events and 80% of patients without events. In each sample, we examined PFS by the same risk stratification approaches noted above and summarized the frequency of p-values <0.05 for the overall comparison and for the ordered pairwise comparisons.

We performed these exercises in two cohorts: 1) the patient cohort used for Figure 5C (SHH) and Figure 6C (G3/G4) which excluded LDCSI (SHH and G3/G4) and carboplatin (G3/G4) subjects; and 2) all patients without excluding LDCSI and carboplatin subjects consistent with the Supplemental Figure 6 (for G3/G4).

As expected, the smaller sample size in the SHH cohort makes it more vulnerable to the variation introduced by the bootstrapping procedure, though the results remain largely consistent. A similar pattern is observed in the G3/G4 cohort with higher consistency seen in the SR vs HR and HR vs VHR groups. Also as expected, risk stratification between arms is less robust and therefore risk classifications that use more than 2 categories have more variability when it comes to ordered pairwise comparisons. However, the overarching clinical benefit of using more than 2 categories is that more patients can be more safely treated with less toxic more effective regimens. Hence, looking at the totality of the results, our risk classification approach performs more favorably than others published previously.

SHH: *Excluding LDCSI ala Figure 5C (results below excluded patients that received LDCSI (all were enrolled on ACNS0331)*

rCM approach

	Overall	LR vs. AR	AR vs. VHR
# of p-values <0.05 (%)			
Sample 80% without replacement	1000 (100%)	685 (68.5%)	899 (89.9%)
Sample 100% with replacement	1000 (100%)	517 (51.7%)	665 (66.5%)

SJMB03 approach

	Overall (=LR vs. VHR)
# of p-values <0.05 (%)	
Sample 80% without replacement	1000 (100%)
Sample 100% with replacement	1000 (100%)

PNET5 approach

	Overall (=LR vs. VHR)
# of p-values <0.05 (%)	
Sample 80% without replacement	1000 (100%)
Sample 100% with replacement	1000 (100%)

PNET5 + TP53 approach

	Overall (=LR vs. VHR)
# of p-values <0.05 (%)	
Sample 80% without replacement	1000 (100%)
Sample 100% with replacement	1000 (100%)

SHH: *Using all available data (including LDCSI patients)*

rCM approach

	Overall	LR vs. AR	AR vs. VHR
# of p-values <0.05 (%)			
Sample 80% without replacement	1000 (100%)	729 (72.9%)	996 (99.6%)
Sample 100% with replacement	1000 (100%)	533 (53.3%)	877 (87.7%)

SJMB03 approach

	Overall (=LR vs. VHR)
# of p-values <0.05 (%)	
Sample 80% without replacement	1000 (100%)
Sample 100% with replacement	1000 (100%)

PNET5 approach

	Overall (=LR vs. VHR)
# of p-values <0.05 (%)	
Sample 80% without replacement	1000 (100%)
Sample 100% with replacement	1000 (100%)

PNET5 + TP53 approach

	Overall (=LR vs. VHR)
# of p-values <0.05 (%)	
Sample 80% without replacement	1000 (100%)
Sample 100% with replacement	1000 (100%)

G3/G4: *Excluding LDCSI and Carboplatin (results below excluded patients randomized to LDCSI (all were enrolled on ACNS0331) or Carboplatin (all were enrolled on ACNS0332))*

CM approach

	Overall	LR vs. SR	SR vs. HR	HR vs. VHR
# of p-values <0.05 (%)				
Sample 80% without replacement	1000 (100%)	1000 (100%)	161 (16.1%)	1000 (100%)
Sample 100% with replacement	1000 (100%)	940 (94%)	165 (16.5%)	992 (99.2%)

rCM approach

	Overall	LR vs. SR	SR vs. HR	HR vs. VHR
# of p-values <0.05 (%)				
Sample 80% without replacement	1000 (100%)	771 (77.1%)	987 (98.7%)	1000 (100%)
Sample 100% with replacement	1000 (100%)	552 (55.2%)	861 (86.1%)	995 (99.5%)

SJMB03 approach

	Overall	Low vs. Int	Int vs. High
# of p-values <0.05 (%)			
Sample 80% without replacement	1000 (100%)	139 (13.9%)	1000 (100%)
Sample 100% with replacement	1000 (100%)	162 (16.2%)	1000 (100%)

Continuum approach

	Overall	High G3 vs. Low G3	Low G3 vs. G3.5	G3.5 vs. Low G4	Low G4 vs. High G4
# of p-values <0.05 (%)					
Sample 80% without replacement	1000 (100%)	171 (17.1%)	0 (0%)	189 (18.9%)	0 (0%)
Sample 100% with replacement	997 (99.7%)	157 (15.7%)	0 (0%)	176 (17.6%)	1 (0.1%)

G3/G4: *Using all available data (including LDCSI and patients that received carboplatin)*

CM approach

	Overall	LR vs. SR	SR vs. HR	HR vs. VHR
# of p-values <0.05 (%)				
Sample 80% without replacement	1000 (100%)	1000 (100%)	4 (0.4%)	1000 (100%)
Sample 100% with replacement	1000 (100%)	998 (99.8%)	8 (0.8%)	1000 (100%)

rCM approach

	Overall	LR vs. SR	SR vs. HR	HR vs. VHR
# of p-values <0.05 (%)				
Sample 80% without replacement	1000 (100%)	984 (98.4%)	993 (99.3%)	1000 (100%)
Sample 100% with replacement	1000 (100%)	851 (85.1%)	915 (91.5%)	977 (97.7%)

SJMB03 approach

	Overall	Low vs. Int	Int vs. High
# of p-values <0.05 (%)			
Sample 80% without replacement	1000 (100%)	404 (40.4%)	1000 (100%)
Sample 100% with replacement	1000 (100%)	289 (28.9%)	1000 (100%)

Continuum approach

	Overall	High G3 vs. Low G3	Low G3 vs. G3.5	G3.5 vs. Low G4	Low G4 vs. High G4
# of p-values <0.05 (%)					
Sample 80% without replacement	1000 (100%)	7 (0.7%)	0 (0%)	548 (54.8%)	0 (0%)
Sample 100% with replacement	990 (99%)	31 (3.1%)	2 (0.2%)	376 (37.6%)	7 (0.7%)

Comparison of the proposed risk stratification vs. other similar models:

We also compared our proposed model to previously considered risk-stratification models by examining Harrell's concordance statistics and AUCs/ROC curves. The risksetROC package in R was used to compute areas under the curve (AUC) of receiver operating characteristic (ROC) curves for 5-year progression-free survival. As the results below demonstrate, our proposed model has slightly better performance compared to the others.

Comparison of **SHH risk stratification** models (excludes LDCSI patients as in Figure 5C)

	**Using all available patients**			**Complete cases^**			
Method[#]	# of patients in model	# of events	Concordance (Harrell's Concordance Statistic (SE))	# of patients in model	# of events	Concordance (Harrell's Concordance Statistic (SE))	AUC^
SJMB03	127	46	0.7283 (0.0243)	125	45	0.7321 (0.0244)	0.7691
PNET5	134	47	0.7066 (0.0310)	125	45	0.6973 (0.0317)	0.6806
PNET5 + TP53	128	45	0.7681 (0.0232)	125	45	0.7626 (0.0235)	0.7875
rCM	127	46	0.7694 (0.0260)	125	45	0.7748 (0.0259)	0.7966

^ Based on n=125 patients with information available to fully classify risk for all 4 risk-stratification approaches; AUC at 5-years

Comparison of **G3/G4 risk stratification** models

	**Using all available patients**			**Complete cases^**			
Method	# of patients in model	# of events	Concordance (Harrell's Concordance Statistic (SE))	# of patients in model	# of events	Concordance (Harrell's Concordance Statistic (SE))	AUC^
CM approach	616	178	0.698 (0.018)	591	172	0.695 (0.019)	0.681
Continuum approach	604	175	0.620 (0.020)	591	172	0.623 (0.021)	0.605
SJMB03 approach	614	178	0.681 (0.016)	591	172	0.679 (0.016)	0.672
rCM approach	604	175	0.719 (0.017)	591	172	0.713 (0.018)	0.698

^ Based on n=591 patients with information available fully classify risk for all 4 risk-stratification approaches; AUC at 5-years
